# Binding of carbon dioxide and acetylene to free carboxylic acid sites in a metal–organic framework†

**DOI:** 10.1039/d4sc00101j

**Published:** 2024-04-10

**Authors:** Christopher Marsh, Xue Han, Zhenzhong Lu, Ivan da Silva, Yongqiang Cheng, Luke L. Daemen, Sarah J. Day, Stephen P. Thompson, Anibal J. Ramirez-Cuesta, Sihai Yang, Martin Schröder

**Affiliations:** a Department of Chemistry, University of Manchester Manchester M13 9PL UK Sihai.Yang@manchester.ac.uk M.Schroder@manchester.ac.uk; b College of Chemistry Beijing Normal University Beijing 100875 China; c ISIS Neutron and Muon Source, Rutherford Appleton Laboratory Oxford OX11 0QX UK; d Neutron Scattering Division, Neutron Sciences Directorate, Oak Ridge National Laboratory Oak Ridge TN 37831 USA; e Diamond Light Source Harwell Science Campus Oxford OX11 0DE UK; f Beijing National Laboratory for Molecular Sciences, College of Chemistry and Molecular Engineering, Peking University Beijing 100871 China Sihai.Yang@pku.edu.cn

## Abstract

The functionalisation of organic linkers in metal–organic frameworks (MOFs) to improve gas uptake is well-documented. Although the positive role of free carboxylic acid sites in MOFs for binding gas molecules has been proposed in computational studies, relatively little experimental evidence has been reported in support of this. Primarily this is because of the inherent synthetic difficulty to prepare MOF materials bearing free, accessible –COOH moieties which would normally bind to metal ions within the framework structure. Here, we describe the direct binding of CO_2_ and C_2_H_2_ molecules to the free –COOH sites within the pores of MFM-303(Al). MFM-303(Al) exhibits highly selective adsorption of CO_2_ and C_2_H_2_ with a high selectivity for C_2_H_2_ over C_2_H_4_. *In situ* synchrotron X-ray diffraction and inelastic neutron scattering, coupled with modelling, highlight the cooperative interactions of adsorbed CO_2_ and C_2_H_2_ molecules with free –COOH and –OH sites within MFM-303(Al), thus rationalising the observed high selectivity for gas separation.

## Abstract 2

Metal–organic frameworks incorporating free carboxylic acid and hydroxyl groups are beneficial for selective uptake of small molecules such as CO_2_ and C_2_H_2_. This has been revealed through SXPD and INS/DFT studies.

## Graphical abstract

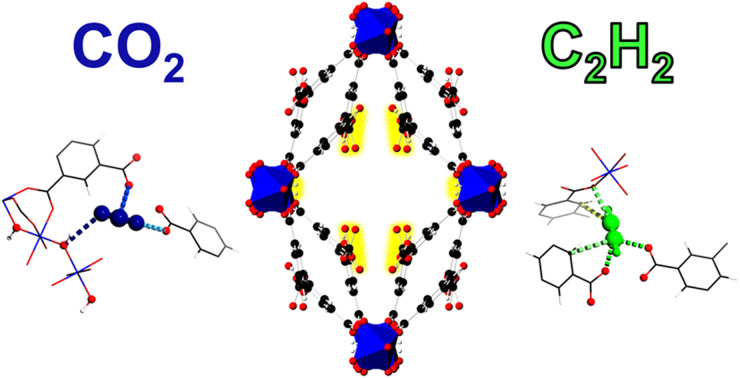

Porous metal–organic framework (MOF) materials have received much interest in the search for sorbent materials for a variety of gases.^1–3^ A commonly employed strategy to obtain MOFs with desirable affinity for target gases is to introduce functional groups to the linker, which subsequently line the pore interior and provide binding sites for gas molecules. State-of-the-art studies primarily focus on groups such as halide,^4^ amide,^5,6^ hydroxyl^7,8^ and amine^9^ groups since they can be incorporated within the protocol for MOF synthesis and the resultant materials often retain their framework structure with the functional group(s) protruding into the pores. Carboxylic acid groups (–COOH), incorporating both lone pairs of electrons and acidic protons, possess high propensity for gases such as CO_2_, as has been suggested by computational studies.^10–12^ However, due to the preference of carboxylate groups to bind to metal centres, there are only a few reports of porous MOFs that are functionalised with free carboxylic acid sites.^13–20^ To date, the molecular details of interactions of carboxylic acid moieties in MOFs with gas molecules have been poorly explored by experiment. Here we report the direct visualisation of binding of CO_2_ and C_2_H_2_ molecules to the free carboxylic acid sites in MFM-303(Al) by a combination of *in situ* synchrotron X-ray powder diffraction (SXPD), inelastic neutron scattering (INS) and density functional theory (DFT) calculations. These crystallographic and dynamic experiments confirm unambiguously the important role of –COOH and –OH sites in assembling a series of cooperative supramolecular interactions with adsorbed CO_2_ and C_2_H_2_ molecules, leading to excellent adsorption properties.

MFM-303(Al) was prepared by hydrothermal reaction of biphenyl-3,3′,5,5′-tetracarboxylic acid (H_4_L) with aluminium chloride in acidified water (pH < 2).^21^ The material, [Al(OH)(H_2_L)], is composed of octahedrally-coordinated Al(iii) centres, with two bridging hydroxyl groups in *trans* positions, and each ligand has two –COOH groups that are not bound to Al(iii) centres and remain protonated. This is promoted by the use of low pH conditions during the synthesis, with the potential of the carboxylic group interacting with guest molecules within the pores *via* hydrogen bonding. The structure of the as-synthesised material thus reveals intramolecular hydrogen bonding between neighbouring carboxylic acid groups [O⋯O = 2.637(14) Å], which helps to stabilise the overall structure. This confirms the approach to overcome the inherent preference of carboxylic acid groups to bind to metal centres *via* the use of strongly acidic conditions combined with multitopic ligands that can support hydrogen bonding. Along the crystallographic *c* axis the widest aperture is ∼8 Å, and the –COOH groups taking van der Waals radii into account are separated by *ca* 2.3 Å (Fig. 1). The phase purity of bulk samples of MFM-303(Al) was confirmed by PXRD (Fig. S1†). Desolvated MFM-303(Al) shows a surface area of 724 m^2^ g^−1^ (Fig. S4†).

**Fig. 1:**
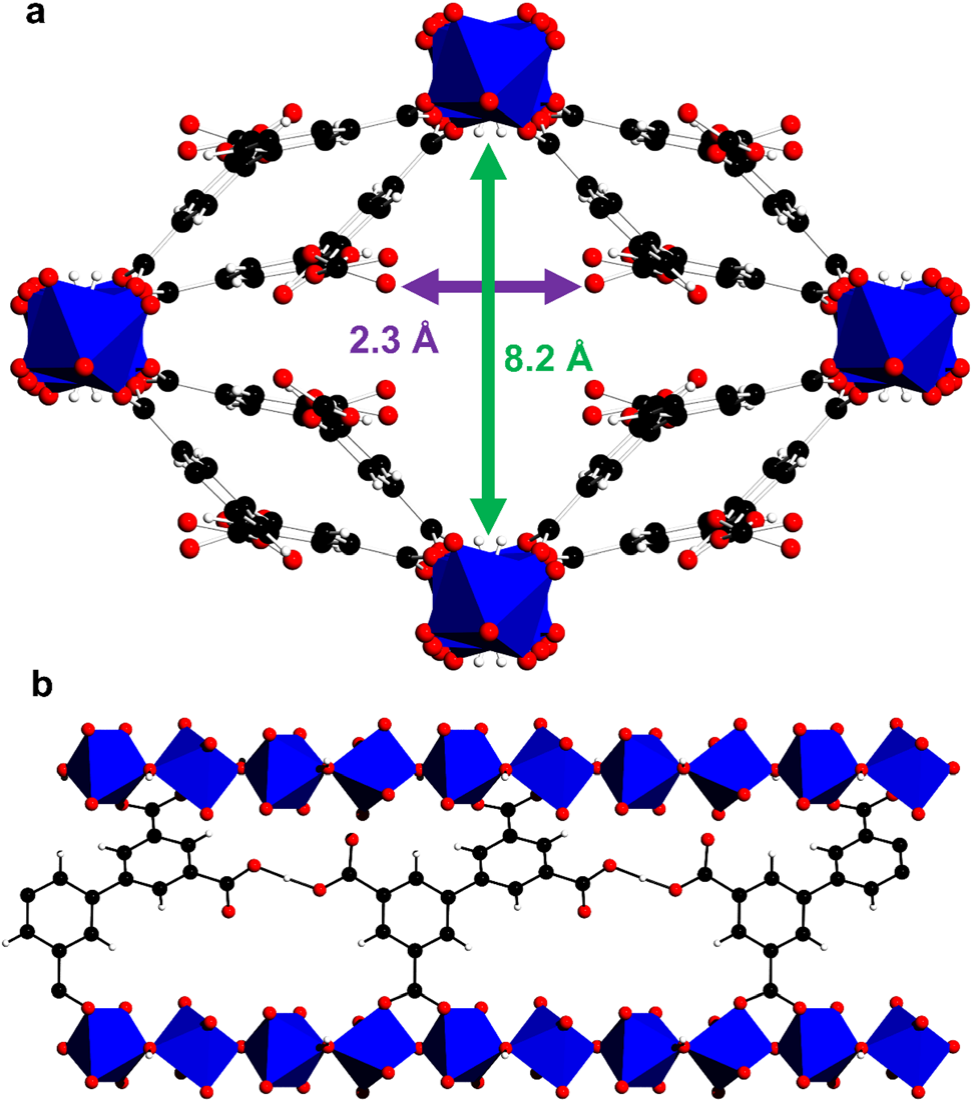
Crystal structure of as-synthesised MFM-303(Al) (water in the pores have been removed for clarity). (a) View along *c* axis, and (b) view along *a* axis showing intramolecular hydrogen bonding between neighbouring free –COOH groups (aluminium, blue; carbon, black; oxygen, red; hydrogen, white; [AlO_4_(OH)_2_], blue octahedra).

CO_2_ isotherms were recorded over a wide temperature range (273–333 K) and showed adsorption–desorption hysteresis, which increased with increasing temperature (Fig. 2a). This is in contrast to many previously reported MOFs, in which hysteresis loops are enhanced at low temperature,^22,23^ and implies that MFM-303(Al) has a small degree of temperature/guest-dependant framework flexibility. The isosteric heat (*Q*_st_) and entropy (Δ*S*) of adsorption of CO_2_ were calculated using the van't Hoff equation (Fig. 2b). The value of *Q*_st_ decreases from 37 to 33 kJ mol^−1^ with CO_2_ loading up to 0.5 mmol g^−1^ and then increases to 36 kJ mol^−1^ between 0.5 and 2.5 mmol g^−1^. This indicates that the strong binding sites are occupied early on initial uptake of CO_2_ and then notable CO_2_⋯CO_2_ interactions are generated with increasing surface coverage. The value of Δ*S* decreases continuously with increasing loading, indicating an increase in local order of the system, again consistent with the formation of CO_2_⋯CO_2_ interactions at higher loadings.

**Fig. 2:**
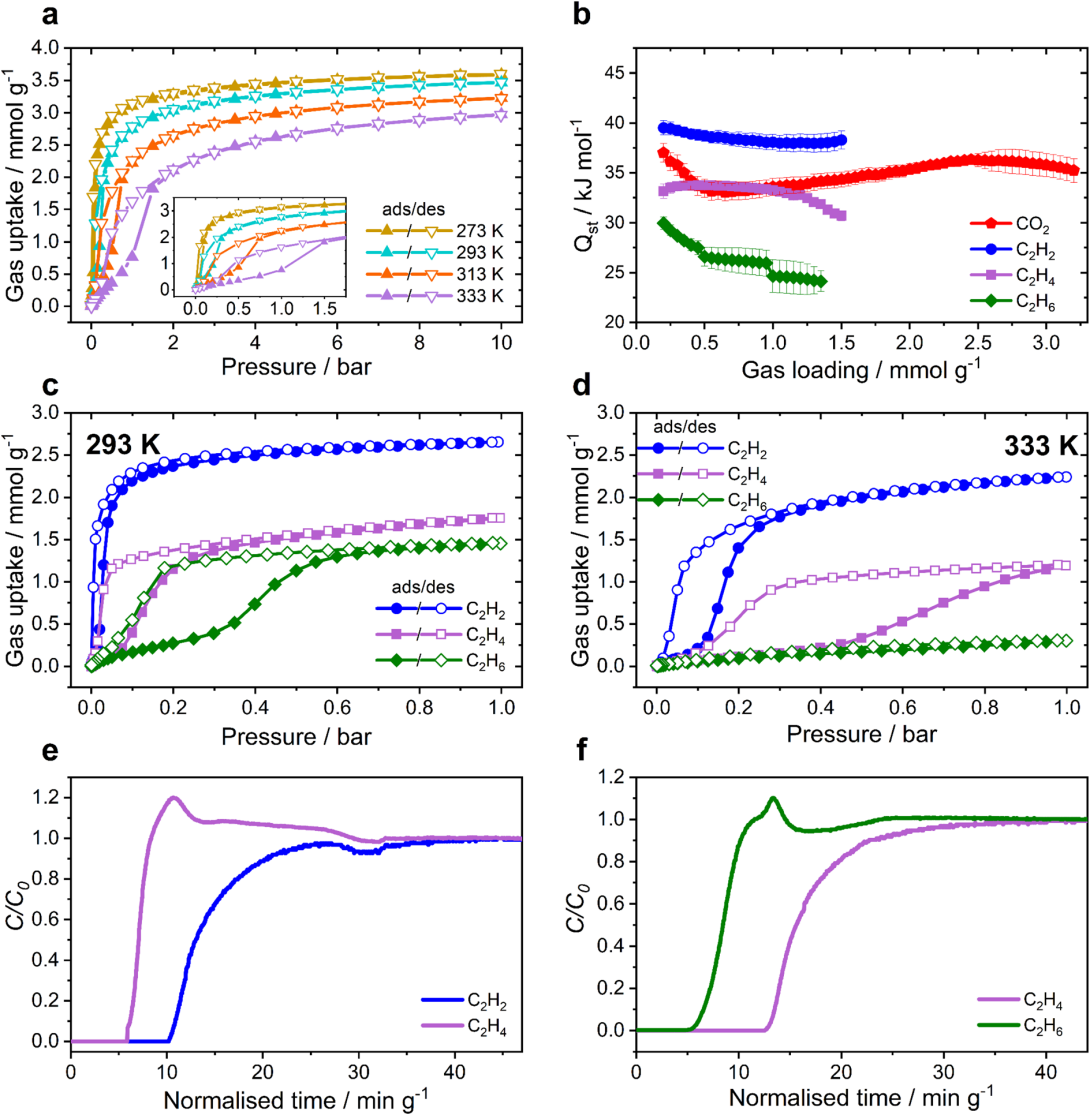
Adsorption and breakthrough data for MFM-303(Al). (a) CO_2_ sorption isotherms recorded from 273–333 K, with inset showing the low pressure region, highlighting the presence of a hysteresis loop with increasing temperature; (b) isosteric heat of adsorption (*Q*_st_) calculated from isotherms using the van't Hoff equation; adsorption isotherms for C_2_ hydrocarbons in MFM-303(Al) at (c) 293 K and (d) 333 K; breakthrough plots for 50 : 50 mixtures of hydrocarbons (e) C_2_H_2_/C_2_H_4_ (f) C_2_H_4_/C_2_H_6_ over a fixed-bed packed with MFM-303(Al) at 333 K and 1 bar (sample mass = 2.33 g; total flow rate = 4 mL min^−1^).

Pores with restricted geometry or with narrow apertures have been shown to have high potential in the field of molecular separation, particularly where the components of mixtures have similar molecular properties, such as sizes and volatilities.^24^ Isotherms for C_2_ hydrocarbons were recorded on MFM-303(Al) at 273–333 K. Hysteresis loops are present for all the C_2_ hydrocarbons and become more prominent at higher temperature (Fig. 2c and d). For C_2_H_2_, an uptake of 2.65 mmol g^−1^ is observed at 293 K and 1 bar, which decreases to 2.24 mmol g^−1^ at 333 K. The hysteresis loop becomes prominent at temperatures above 313 K. For C_2_H_4_, a lower uptake of 1.75 mmol g^−1^ was measured at 293 K and 1 bar, decreasing to 1.19 mmol g^−1^ at 333 K. The hysteresis loop is much more pronounced than for C_2_H_2_, being apparent for measurements at all temperatures. Meanwhile, an uptake for C_2_H_6_ of 1.45 mmol g^−1^ at 293 K and 1 bar was observed, reducing to 0.30 mmol g^−1^ at 333 K. The values for *Q*_st_ for C_2_H_2_, C_2_H_4_ and C_2_H_6_ were measured to be 38–42, 34–32 and 30–24 kJ mol^−1^, respectively, the order of *Q*_st_ values being consistent with the observed uptake capacities and inversely related to the magnitude of the observed hysteresis. This suggests that the adsorbates with the largest molecular size do not enter the framework as readily and once inside become trapped in the pore, which results in a hysteresis loop and a lower value of *Q*_st_ but higher Δ*S* (Fig. S8†). Adsorption of CH_4_ shows broad hysteretic loops and much lower uptakes (Fig. S5†).

The presence of hysteresis loops in the isotherms suggests that there is a possible structural change from the activated to the guest-loaded material. The magnitude of the hysteresis loop is inversely proportional to the observed values of *Q*_st_ with more strongly interacting adsorbates having a narrower loop. This suggests that the structural change can be modulated by the choice of guest, and molecules with stronger interactions can more readily overcome the energy barrier to re-organisation.^25^ Also, for the C_2_ hydrocarbons, the uptake capacities and values of *Q*_st_ are inversely proportional to the kinetic diameter of the adsorbate (Table S3†). The hysteresis loops are dependent on temperature, becoming wider at higher temperature, consistent with other MOFs that feature guest-dependent flexibility;^26^ this is in contrast to capillary condensation in rigid porous structures.^27^ Within MFM-303(Al), the intramolecular hydrogen bonding can reorientate from that in the activated empty structure in order to optimise interaction of the –COOH moieties with guest molecules. The presence of flexibility in sorbent materials is often beneficial to gas separations, and materials can breathe and/or undergo a gate-opening response dependent on the size and shape of the molecule. The pressure under which structural transformation occurs is unique for each adsorbent, and thus excellent separation performance and selective uptake of target molecules can be achieved.^28,29^

The adsorption selectivity of MFM-303(Al) for equimolar binary mixtures was estimated using ideal adsorbed solution theory (IAST) based upon single-component isotherms.^30,31^ Interestingly, MFM-303(Al) exhibits an excellent IAST selectivity of 15.0 for C_2_H_2_ over C_2_H_4_ at 1 bar and 293 K (Fig. S11†). This compares well with other MOFs in the literature. For example, at 298 K, MOF-74(Fe) possesses a selectivity of 2.08 (ref. 32) whilst UTSA-100 achieves 10.72 (ref. 33) and NbU-1 reaches 5.9.^34^ The excellent selectivity is corroborated by comparison to other reported MOFs containing acidic –OH and –COOH groups, such as Ni-gallate^35^ and NTU-72 (ref. 36) which show exceptional selectivities of 43.7 and 56 for 1 : 99 mixtures, though in these examples their potential for other mixtures of hydrocarbons was not fully explored. A high selectivity for CO_2_/CH_4_ is also observed for MFM-303(Al) (56.1 at 293 K and 1 bar), which again compares well with other MOFs (Table S2†). Thus, the restricted pores of MFM-303(Al) lead to enhanced selectivity for C_2_H_2_ and CO_2_. Dynamic breakthrough separation of an equimolar mixture of C_2_H_2_/C_2_H_4_ and C_2_H_4_/C_2_H_6_ was undertaken at 333 K (Fig. 2e and f). Clear separation of components was observed in both cases with selective retention of C_2_H_2_ and C_2_H_4_ demonstrating the potential of MFM-303(Al) as a sorbent material for molecular separation.

Two unique binding domains (I and II) for CO_2_ have been determined using Rietveld refinement of SXPD data for CO_2_-loaded MFM-303(Al) (Fig. 3a–c). Notable interactions are observed between CO_2_ molecules and the free –COOH and bridging –OH sites in the pore. CO_2_^I^ (occupancy of 1.0) is stabilised by moderate hydrogen bonding interactions to the bridging hydroxyl group [μ_2_-*O*H⋯*O*=C=O = 3.21(1) Å] and the free –COOH [O=C=*O*⋯*O* = 2.67(1) Å]. CO^II^_2_ [occupancy of 0.61(1)] shows similar distances to neighbouring –COOH groups [O=C=*O*⋯*O* = 2.75(1) Å]. Furthermore, adsorbed CO_2_ molecules in the pore form the typical “T” shape arrangement [O=C^I^=*O*⋯*C*O_2_^II^ = 2.60(1) Å], suggesting that intermolecular dipole interaction also contributes to the packing of CO_2_ in the pore, consistent with the values for *Q*_st_.

**Fig. 3:**
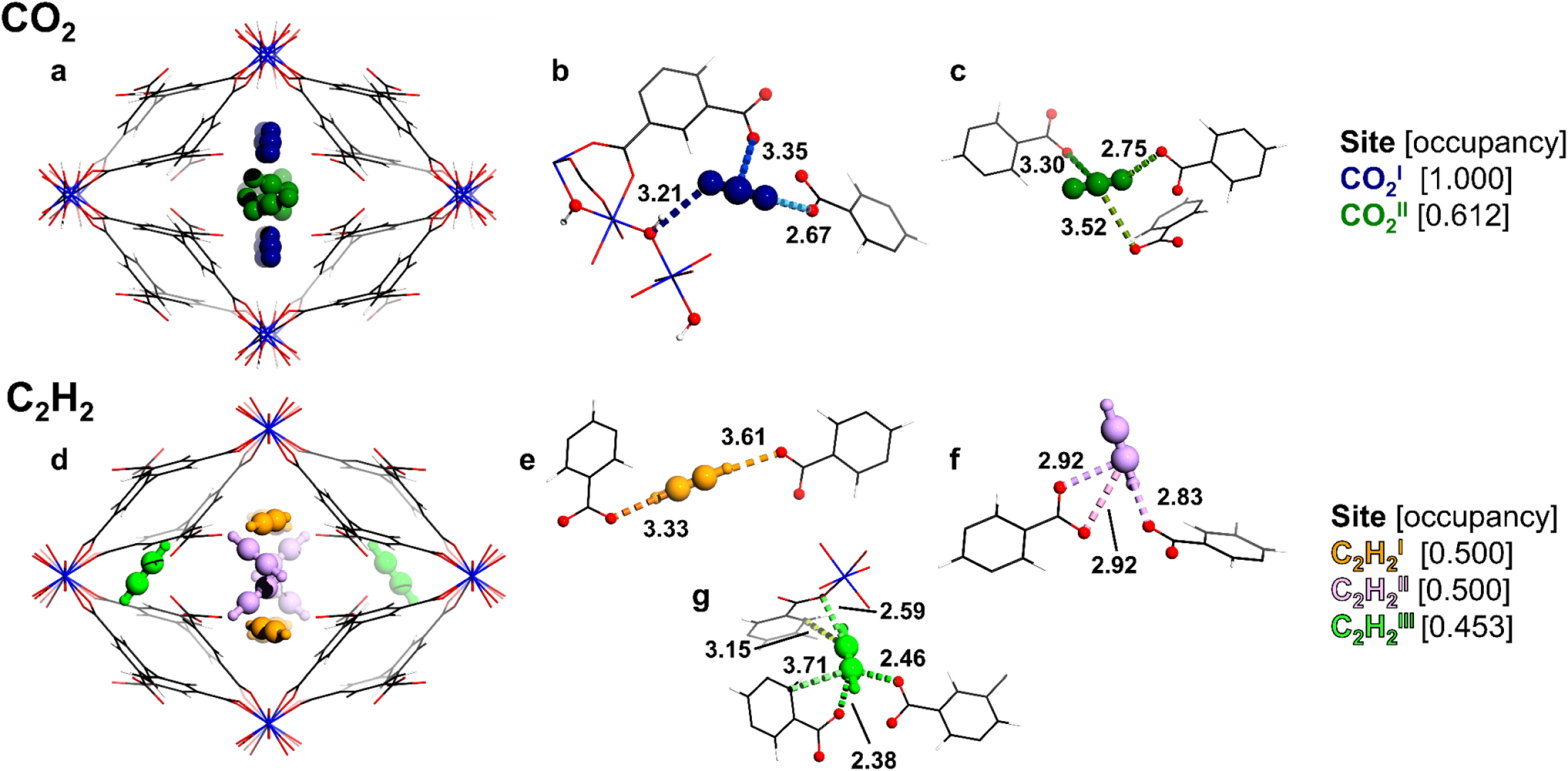
The crystal structure of gas-loaded MFM-303(Al) determined *via* Rietveld refinement of high-resolution SXPD data showing two unique sites for CO_2_ (a–c) and three for C_2_H_2_ (d–f) with partial occupancies (aluminium, blue; carbon, black; oxygen, red; hydrogen, white; CO^I^_2_, dark blue; CO^II^_2_, dark green; C_2_H_2_^I^, orange; C_2_H_2_^II^, pink; C_2_H_2_^III^, light green). Views down the crystallographic *c*-axis: (a) and (d) packing of adsorbed molecules; (b) the interaction of CO_2_^I^ with the free –COOH and μ-OH groups of the framework; (c) medium–strong interactions of CO^II^_2_ with free –COOH groups; (e and f) interactions of C_2_H^I^_2_ and C_2_H_2_^II^ with free –COOH groups; (g) interaction of C_2_H_2_^III^ with free –COOH groups and the phenyl ring of the framework.

In contrast, three binding sites (I–III) were determined for adsorbed C_2_H_2_ molecules in MFM-303(Al) (Fig. 3d–g). C_2_H_2_^I^ [occupancy of 0.50(1)] is located towards the centre of the pore and forms strong hydrogen bonds between adjacent –COOH sites [H–C≡*C*–H⋯*O* = 3.61(5), 3.33(5) Å]. C_2_H_2_^II^ [occupancy of 0.50(1)] is oriented differently, but also interacts with –COOH groups [H–C≡*C*–H⋯*O* = 2.83(4), 3.42(4) Å], suggesting moderate hydrogen-bonding interactions. Finally, C_2_H_2_^III^ [occupancy of 0.45(1)] is in the small pocket at the corner of the pore and is stabilised primarily *via* confinement effects due to the restricted pore geometry, with contacts to coordinated carboxylate groups [H–C≡*C*–H⋯*O* = 2.38(6), 2.46(7) Å] and the phenyl ring [H–C≡*C*–H⋯*C*_phenyl_ = 3.15(5), 3.72(7) Å]. Additionally, strong interactions are found between C_2_H_2_^III^ and nearby carboxylate groups with H–C≡*C*–H⋯*O* distances of 2.59(4) Å. Thus, the free carboxylic acid sites, along with the hydroxyl group, play a crucial role in binding to these guest molecules. Interestingly, the present study represents the first example of observation of binding of CO_2_ and C_2_H_2_ molecules at free –COOH groups within MOFs.

*In situ* INS measurements were conducted for MFM-303(Al) as a function of loading of CO_2_ and of C_2_H_2_ (Fig. 4). The difference spectra were obtained by subtracting the INS spectrum of bare MFM-303(Al) from the spectra of gas-loaded samples. DFT calculations of the INS spectra of both the bare and gas-loaded systems show excellent agreement with experimental data, allowing the full assignment of the vibrational modes. The difference spectrum of the CO_2_-loaded sample showed several changes. Difference peaks I and II are both combinations of vibrational modes, most significantly the out-of-plane wagging of –OH in the –COOH group, along with aromatic C–H asymmetric bending and C–H out-of-plane bending modes. Peak III is identified as a blueshift of the mode at 959–979 cm^−1^, assigned to the bridging –OH group rocking parallel and in the plane of the [AlO_4_(OH)_2_] chain. Peak IV is assigned to a combination of vibrational modes, most significantly the O–H of COOH in-plane wagging and C–O–H deformation, along with a contribution from the aromatic C–H in-plane wagging. For C_2_H_2_, five notable changes are observed in the difference spectrum. Peak I centred at 667 cm^−1^ arises from a combination of modes: the bridging –OH along-chain wagging, C_2_H_2_ bending and aromatic C–H out-of-plane wagging. Peak II is also assigned to a combination of modes, the antisymmetric C–H binding in C_2_H_2_, and the in-plane wagging of the –OH group of the free carboxylic acid group. Peak III at 850 cm^−1^ arises from the O–H of the free COOH in-plane wagging mode, whilst peaks IV and V arise from a combination of the aromatic C–H out-of-plane wagging and the free carboxylic acid group O–H in-plane wagging, respectively. These observations on the host–guest binding dynamics are entirely consistent with the crystallographic models determined by SXPD data for CO_2_- and C_2_H_2_-loaded MFM-303(Al) and further confirm the positive impact of free –COOH sites on gas adsorption in MOFs.

**Fig. 4:**
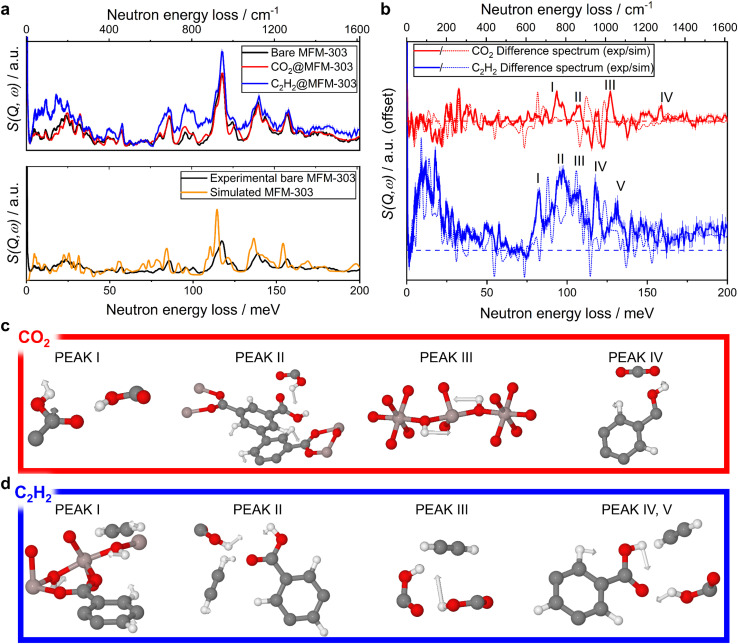
(a) (top) INS spectra of the activated, CO_2_-loaded and C_2_H_2_-loaded MFM-303(Al); (bottom) comparison between INS spectra of MOF using DFT and experimental INS data. (b) Difference spectra between the bare and gas-loaded MFM-303(Al) with key vibrational modes indicated which change in intensity upon gas loading (dashed horizontal line indicates *y* = 0 for each spectrum). (c and d) Visualisation of the vibrational modes that correspond to the indicated peaks in the difference spectrum using DFT-calculated INS spectra. The presence of framework flexibility contributes to the observed discrepancies between the simulated and experimental spectra.

MFM-303(Al), bearing free, accessible –COOH groups and incorporating narrow pores, shows selective uptake of CO_2_ and C_2_H_2_. This selectivity is a result of multiple factors: firstly, the larger value of *Q*_st_ for C_2_H_2_ than C_2_H_4_ shows that MFM-303(Al) has a stronger binding preference, with a lower Δ*S* indicating more ordered pores when loaded with C_2_H_2_. The strong interaction of MFM-303(Al) with CO_2_ and C_2_H_2_ was revealed through *in situ* SXPD and INS/DFT studies with adsorbed C_2_H_2_ and CO_2_ being well-ordered within the structure *via* interaction with the free carboxylic acid and hydroxyl groups, whilst also being confined within the pores. In comparison, C_2_H_4_ shows an overall lower uptake and a weaker interaction with the MOF, related also to its larger kinetic diameter. Interestingly, adsorption of small gas molecules in MFM-303(Al) shows hysteresis, which increases with increasing temperature and molecular size of the gas molecules.

MFM-303(Al) illustrates the importance of the free –COOH group in binding with CO_2_ and C_2_H_2_ molecules, and demonstrates that there is much potential in developing MOFs containing free carboxylic acid sites for selective gas separation. More broadly, the incorporation of functional groups on the interior of MOF materials provides favourable binding interactions with target guest molecules, and is a key strategy for improving selective uptake. This can be combined with optimisation of the pore geometry for the target gas molecules, enabling them to pack efficiently within the pores, thus increasing the overall uptake and selectivity. There is a careful balance to be struck, as efficient packing due to restricted pore geometry and volume may lead to an overall lower uptake when compared with materials with larger pore volumes. Thus, it is important to consider if the intended application is molecular separation (with greater emphasis on selectivity) or gas storage (with greater emphasis on total uptake/packing density). This work also emphasises the power of *in situ* SXPD and INS studies to reveal and understand the nature of host–guest interaction. This deserves further attention in studies of other porous solids showing exceptional adsorption selectivity.

## Data availability

Additional details of experimental methods and procedures, crystallographic and thermodynamic data, isotherms and their fitting and analysis, and of DFT calculations are given in the ESI† for this manuscript.

## Author contributions

CM: synthesis of materials, and their characterisation and analysis. XH, ZL: gas adsorption studies and studies at national facilities. YC, LLD, AJR-C: inelastic neutron scattering experiments, analysis and modelling. IdS: structural analysis and refinement. SJD, SPT: synchrotron X-ray diffraction studies. SY, MS: design, development and supervision of project. Preparation of manuscript with all authors.

## Conflicts of interest

The authors declare no competing interest.

## Supplementary Material

SC-015-D4SC00101J-s001

SC-015-D4SC00101J-s002
