# Supplementary material for: Binding of carbon dioxide and acetylene to free carboxylic acid sites in a metal–organic framework
Source: Chem Sci. 2024 Apr 10;15(21):8197–203. doi: 10.1039/d4sc00101j (PMC11134375; doi:10.1039/d4sc00101j)
Supplement: SC-015-D4SC00101J-s001 [file SC-015-D4SC00101J-s001.pdf]

Electronic Supplementary Material (ESI) for Chemical Science.
This journal is © The Royal Society of Chemistry 2024

Supporting Information

Binding of carbon dioxide and acetylene to free carboxylic acid sites in a metal-organic framework

Christopher Marsh[1], Xue Han[1.2], Zhenzhong Lu[1], Ivan da Silva[3], Yongqiang Cheng[4], Luke L. Daemen[4], Sarah J. Day[5], Stephen P. Thompson[5], Anibal J. Ramirez-Cuesta[4], Sihai Yang[1,6]* and Martin Schröder[1]*

1. Department of Chemistry, University of Manchester, Manchester, M13 9PL, UK
   E-mails: Sihai.Yang@manchester.ac.uk; M.Schroder@manchester.ac.uk
2. College of Chemistry Beijing Normal University, Beijing 100875, China
3. ISIS Neutron and Muon Source, Rutherford Appleton Laboratory, Oxford, OX11 0QX, UK
4. Neutron Scattering Division, Neutron Sciences Directorate, Oak Ridge National Laboratory, Oak Ridge, TN 37831, USA
5. Diamond Light Source, Harwell Science Campus, Oxford, OX11 0DE, UK
6. Beijing National Laboratory for Molecular Sciences, College of Chemistry and Molecular Engineering, Peking University, Beijing 100871, China
   Email: Sihai.Yang@pku.edu.cn

1. Experimental methods and procedures

Chemicals: Aluminum chloride ($AlCl_3$, >99%, Fluka) and hydrochloric acid (HCl, >99%, Fischer Scientific) were used as purchased. Biphenyl-3,3',5,5'-tetracarboxylic acid (H_4L) was synthesised by the literature method.[1]

Methods

Synthesis of [Al(OH)($C_{16}O_8H_8$)](H_2O)$_2$ (MFM-303-solvate):

Biphenyl-3,3',5,5'-tetracarboxylic acid (60 mg, 0.182 mmol) and $AlCl_3$ (121.2 mg, 0.909 mmol) were combined in water (10 mL) acidified with 2% HCl (2 mL) and placed in a PTFE-lined stainless steel autoclave. The autoclave was sealed and heated for 3 days at 483 K. The white crystalline product was isolated by filtration, washed with water and dried in air. Yield: 59.1 mg (79.6% based on ligand). Elemental analysis (% calc/found): $AlO_{11}C_{16}H_{13}$ (C 47.07/46.90, H 3.22/3.32, N 0.0/0.0). Selected IR (ATR): v/cm^{-1}: 3085(w), 1683 (m), 1615 (m), 1579 (s), 1409(m), 1246(m), 1167(m), 1089 (m), 986(s), 803(m), 764(s), 648(m).

Gas adsorption isotherms

Gravimetric isotherms were measured on an IGA system (Hiden Isochema) under ultra-high vacuum in a clean system with a diaphragm and turbo pumping system. All gases were ultra-pure research-grade (99.9999%) and were purchased from BOC and used as received. The as-synthesised MOF was outgassed at 393 K over 18 h prior to measurement.

Calculation of Ideal Adsorbed Solution Theory (IAST) selectivities

The pure component isotherms were fitted using the dual-site Langmuir-Freundlich model, and selectivities (S) calculated using the IAST equation below, where x_i is the amount of each component adsorbed, and y_i is the mole fraction of each component in the gas phase at equilibrium:

$$S = \frac{\left(\frac{x_1}{y_1}\right)}{\left(\frac{x_2}{y_2}\right)}$$

Dynamic gas breakthrough experiments

Breakthrough experiments were carried out in a fixed-bed tube (7 mm diameter, 120 mm length) packed with 2.33 g of MFM-303. The sample was activated by heating under a flow of He for 1 day at 423 K. The fixed bed was cooled to 333 K using a water bath and breakthrough experiments performed using a flow of 1:1 C_2H_2:C_2H_4 or C_2H_4:C_2H_6 at atmospheric pressure with a combined flow rate of 4 mL min^{-1}. The concentration of gases was determined by mass spectrometry and compared with the inlet concentration C_0, where $C/C_0 = 1$ indicates complete breakthrough.

High-resolution synchrotron powder X-ray diffraction and structure determination of binding domains for adsorbed CO_2 and C_2H_2 molecules

High resolution *in situ* synchrotron X-ray powder diffraction (SXPD) data were collected at Beamline I11 of Diamond Light Source using multi-analysing crystal-detectors (MACs) and monochromated radiation (λ = 0.825774 Å). The powder sample was loaded into a capillary tube of 0.7 mm diameter, degassed at 393 K, gas loaded at 298 K and the data collection was carried out at 200 K to minimise the disorder of guest molecules. The sample was reactivated between different gas loadings.

Inelastic neutron scattering

Inelastic neutron scattering (INS) measurements were obtained using the VISION spectrometer at the Spallation Neutron Source, Oak Ridge National Laboratory. The sample was loaded in an aluminium can, degassed at 393 K over 24 h and data collected at <10 K. Gases were dosed at room temperature and the sample cooled to <10 K before data collection.

2. Crystallographic data

Table S1. Summary of crystallographic data for gas-loaded MFM-303

Chemical formula	$C_{16}H_8AlO_9 \cdot 1.61(CO_2)$	$C_{16}H_8AlO_9 \cdot 1.45(C_2H_2)$
M_r	442.15	407.41
Crystal system, space group	Monoclinic, *C*2/*c*	Monoclinic, *C*2/*c*
Temperature (K)	200	200
a, *b*, *c* (Å)	14.24522(16),19.7916(3), 13.0818(2)	14.4863(3), 20.0386(3), 13.2442(2)
β (°)	92.544 (13)	93.0825(17)
V (Å^3)	3654.25	3839.03(11)
Z	8	8
Radiation type	synchrotron, λ = 0.825774(2) Å	
Refinement		
R_{wp}(%)	9.15	9.50
R_{exp}(%)	2.79	3.99
R_p(%)	6.59	6.71
GoF	3.29	2.38

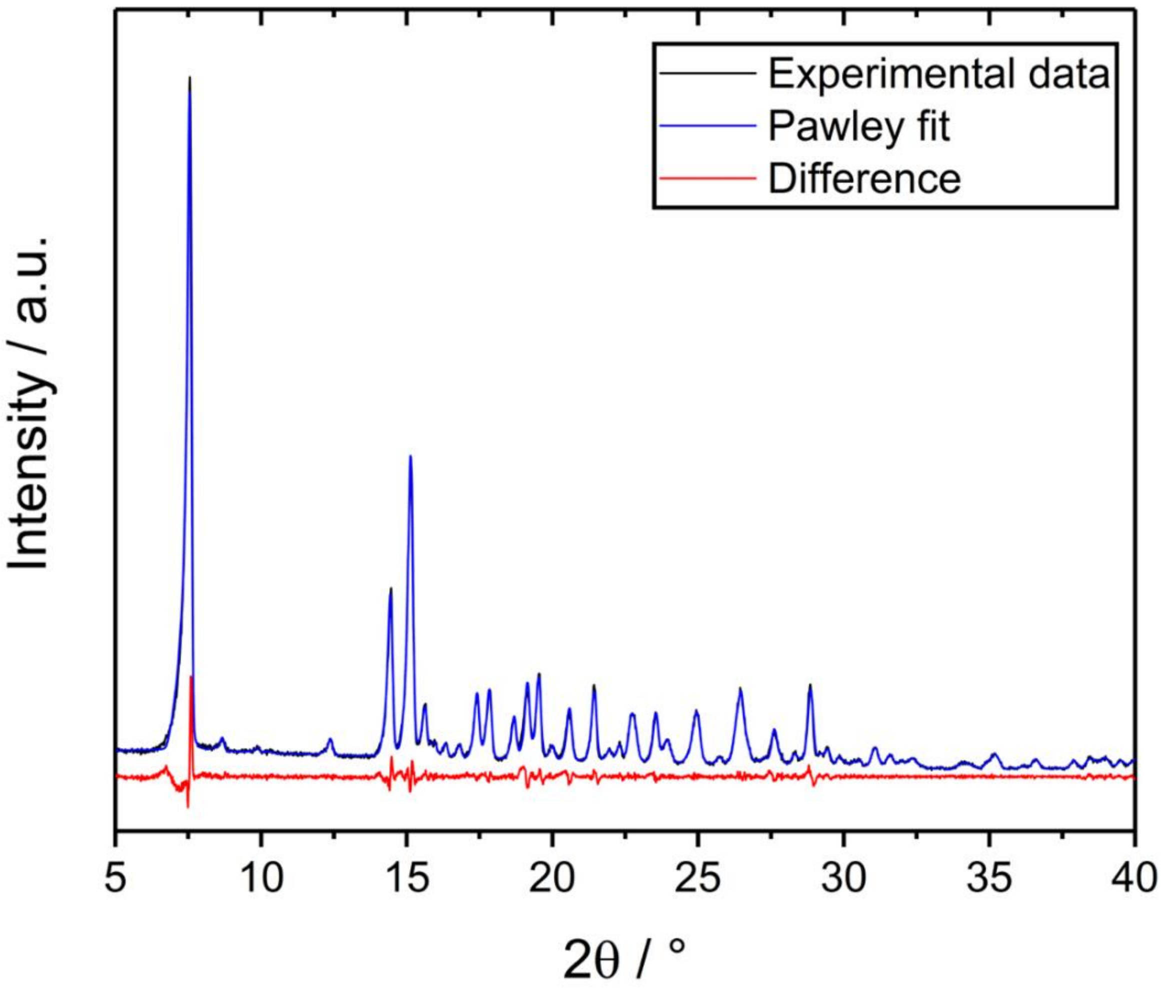

Figure S1. Pawley fitting of experimental MFM-303 bulk sample confirming the phase-purity of the material (λ = 1.5406, R_p=6.280%, R_{wp} = 8.264%, goodness-of-fit = 2.070).

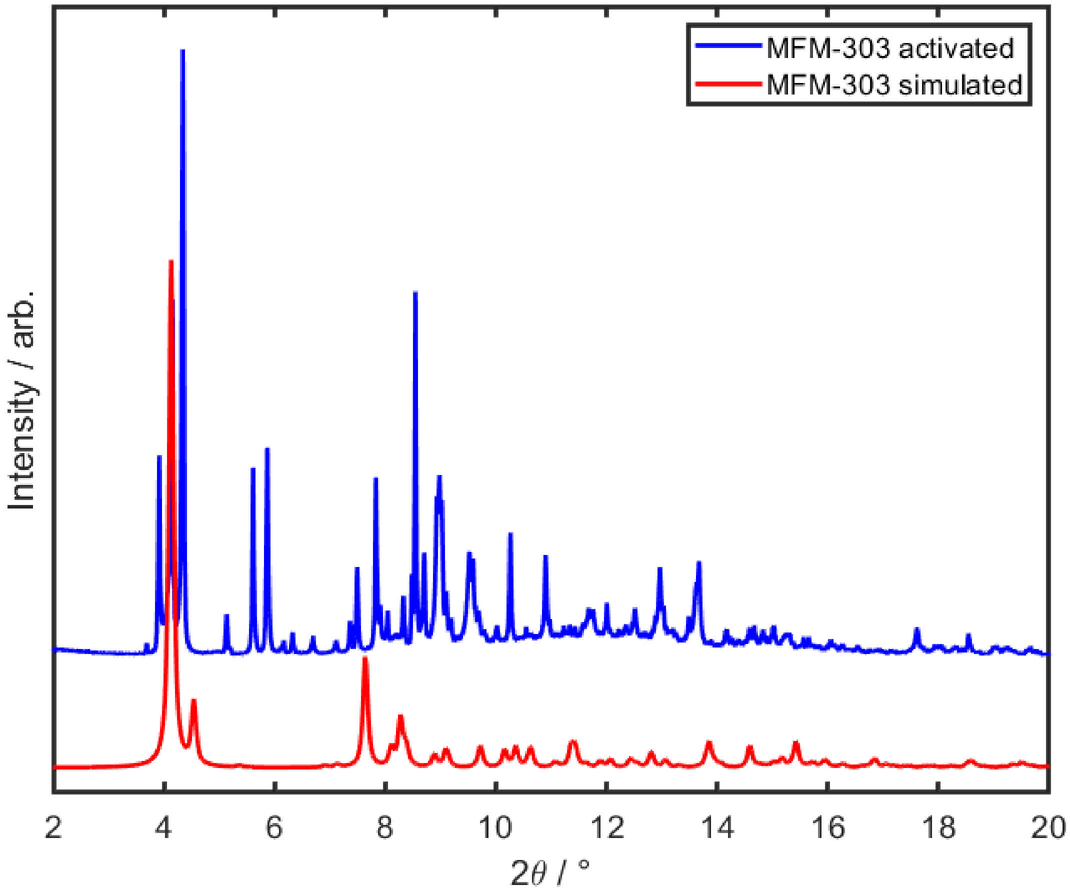

Figure S2. Synchrotron X-ray diffraction data for MFM-303(Al); simulated from the crystal structure of the as-synthesised material and for the *in situ* activated material (λ = 0.825774). Due to the low symmetry of the activated structure with significant overlap of Bragg peaks in a triclinic system, the structure of activated material unfortunately cannot be determined despite our best efforts. However, when guest molecules enter the MOF, the structure returns to its original monoclinic crystal system.

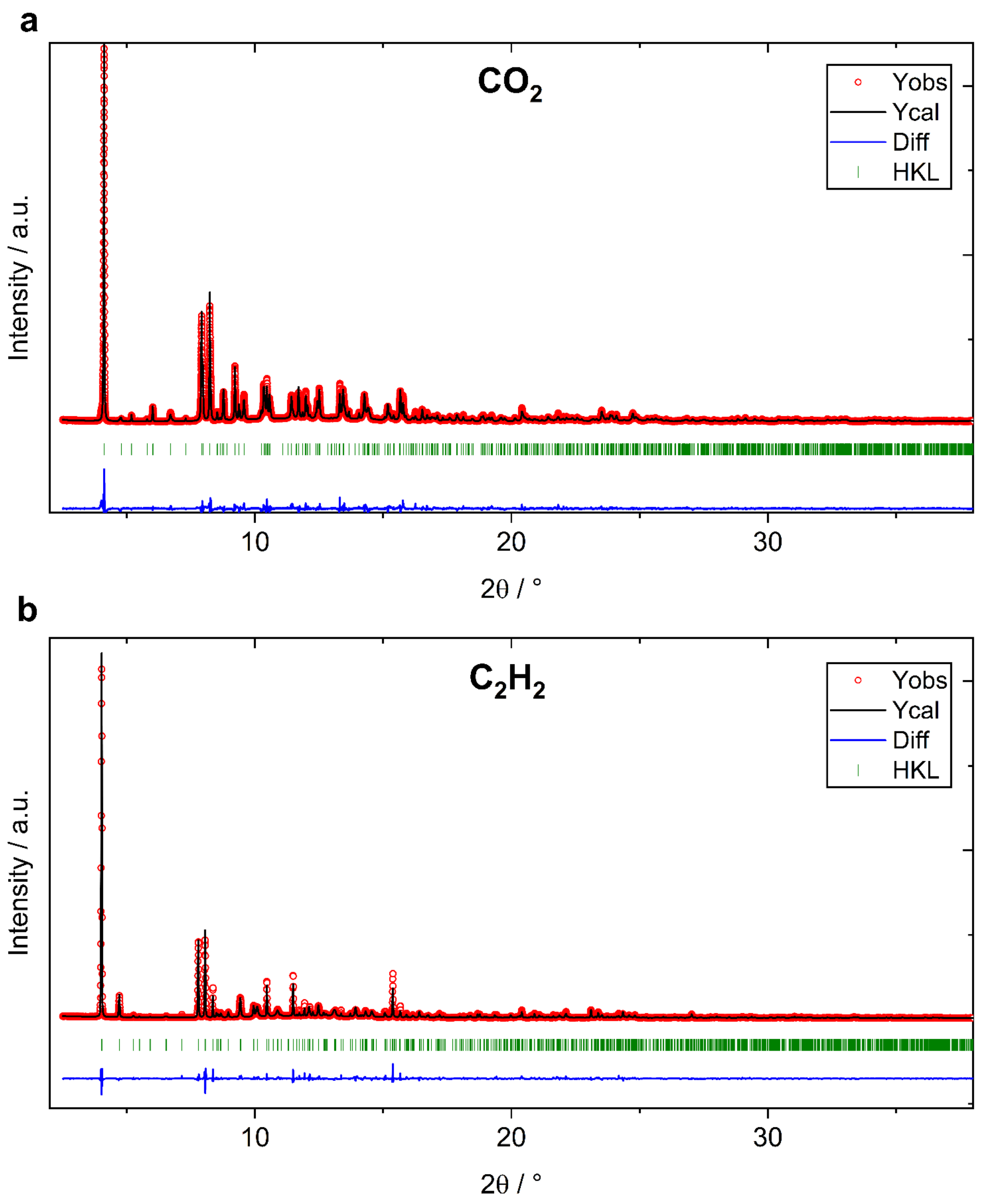

Figure S3. Rietveld refinement for (a) CO_2@MFM-303(Al); (b) C_2H_2@MFM-303(Al).

3. Isotherms

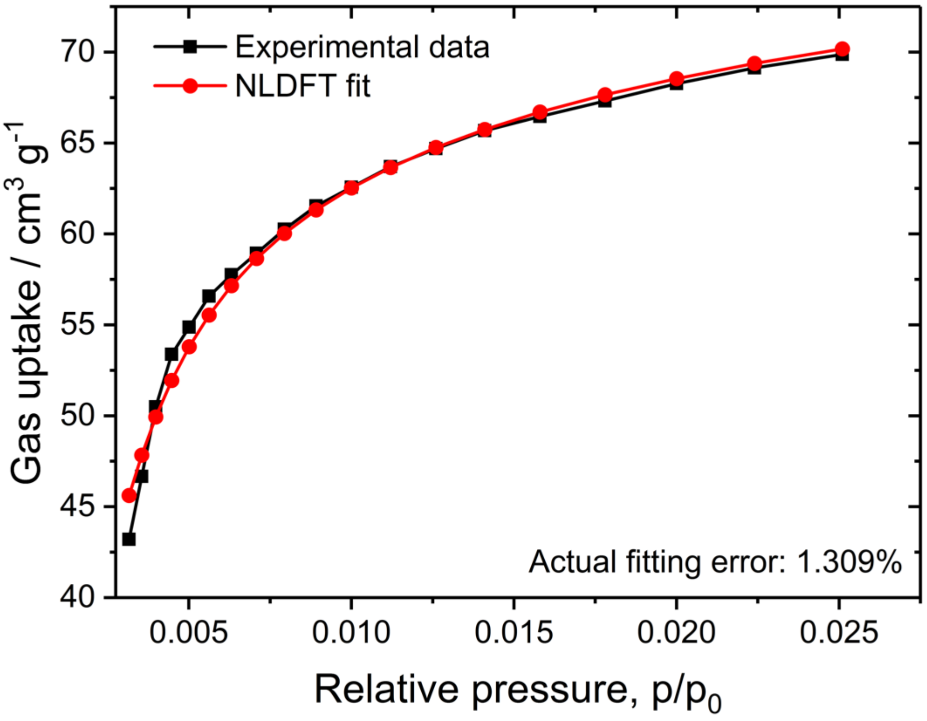

Figure S4. N_2 isotherm for MFM-303(Al) at 77 K with NLDFT fitting used to calculate the surface area of 724 m^2 g^{-1}.

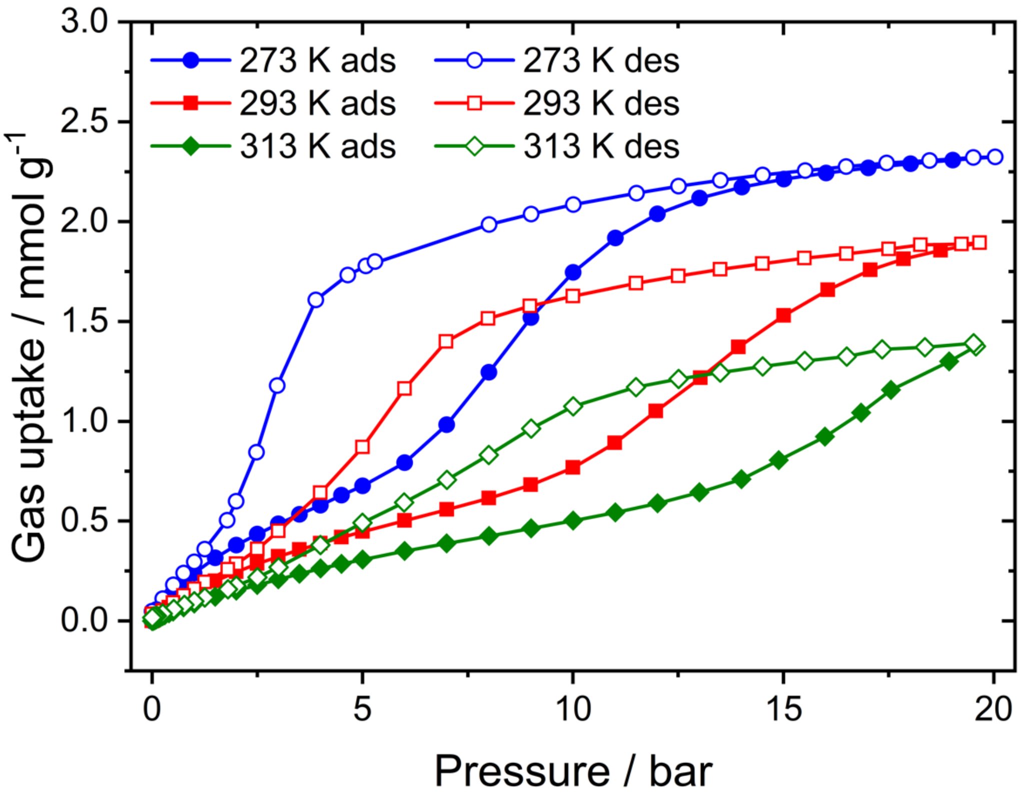

Figure S5. CH_4 isotherms for MFM-303(Al) collected at various temperatures up to 20 bar. It was found that the uptake was considerably lower than for any of the other hydrocarbons measured, with 0.15 mmol g^{-1} CH_4 adsorbed at 293 K, 1 bar.

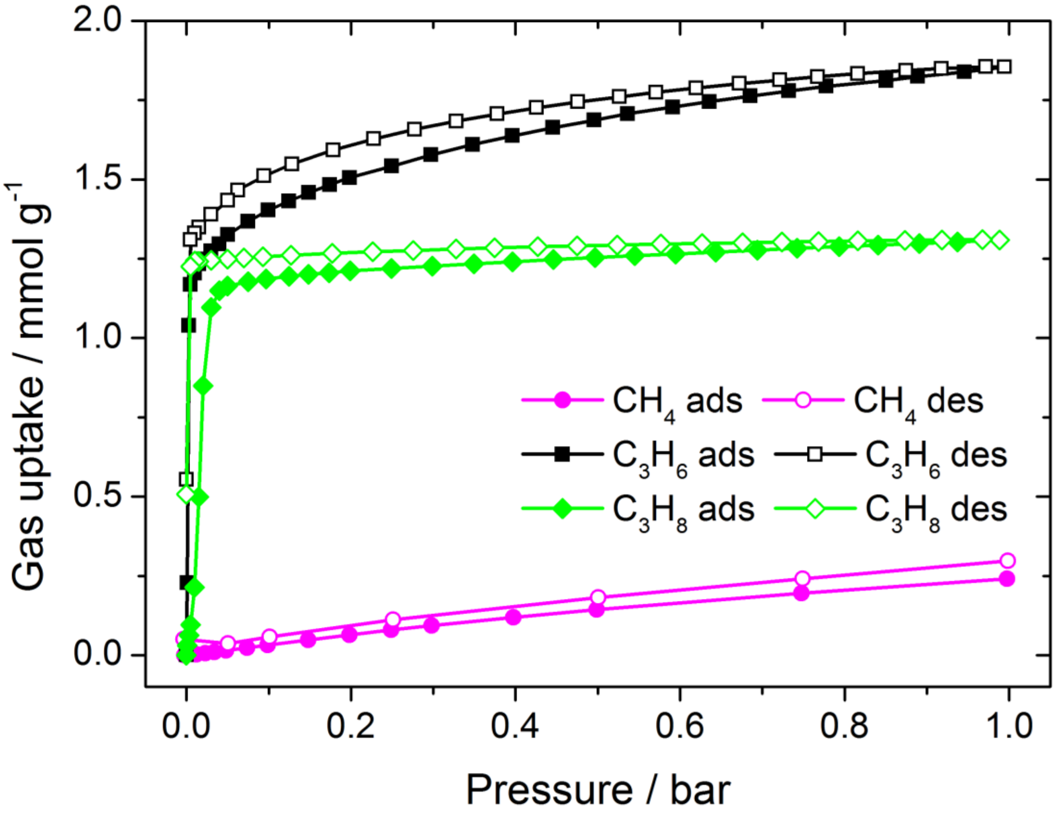

Figure S6. CH_4 and C_3 hydrocarbon isotherms for MFM-303(Al) collected at 293 K, up to 1 bar.

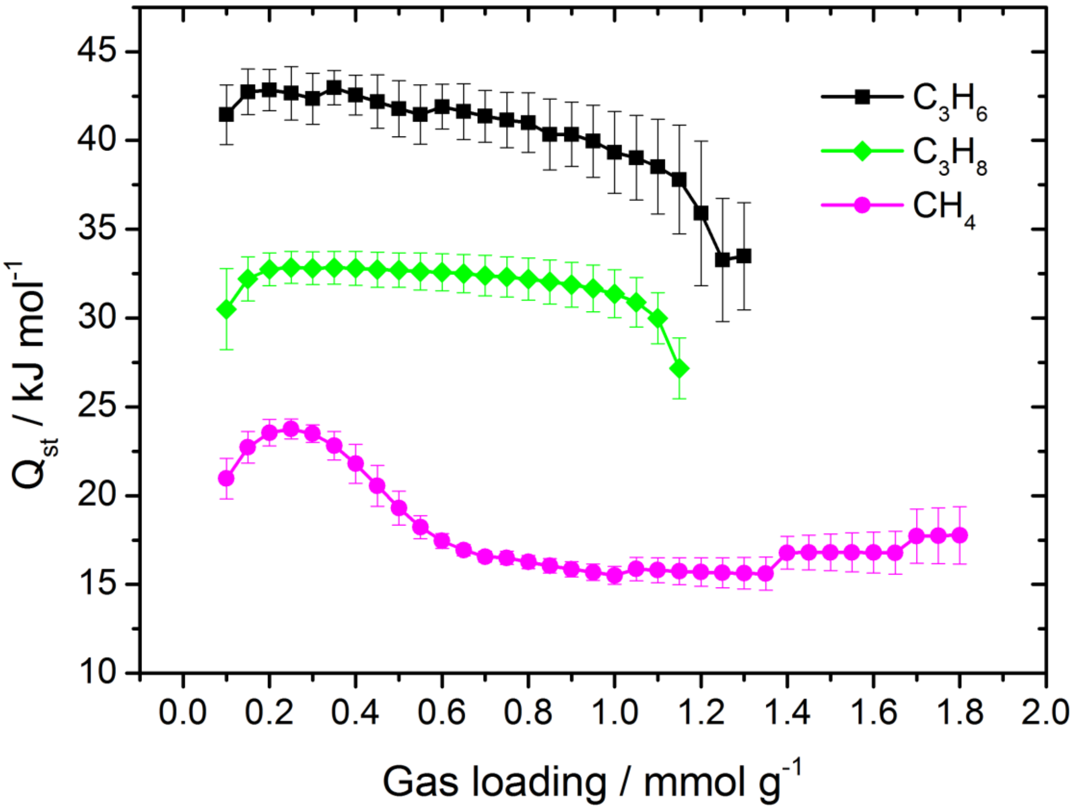

Figure S7. Isosteric heat of adsorption for CH_4 and C_3 hydrocarbon isotherms for MFM-303(Al).

4. Thermodynamic data

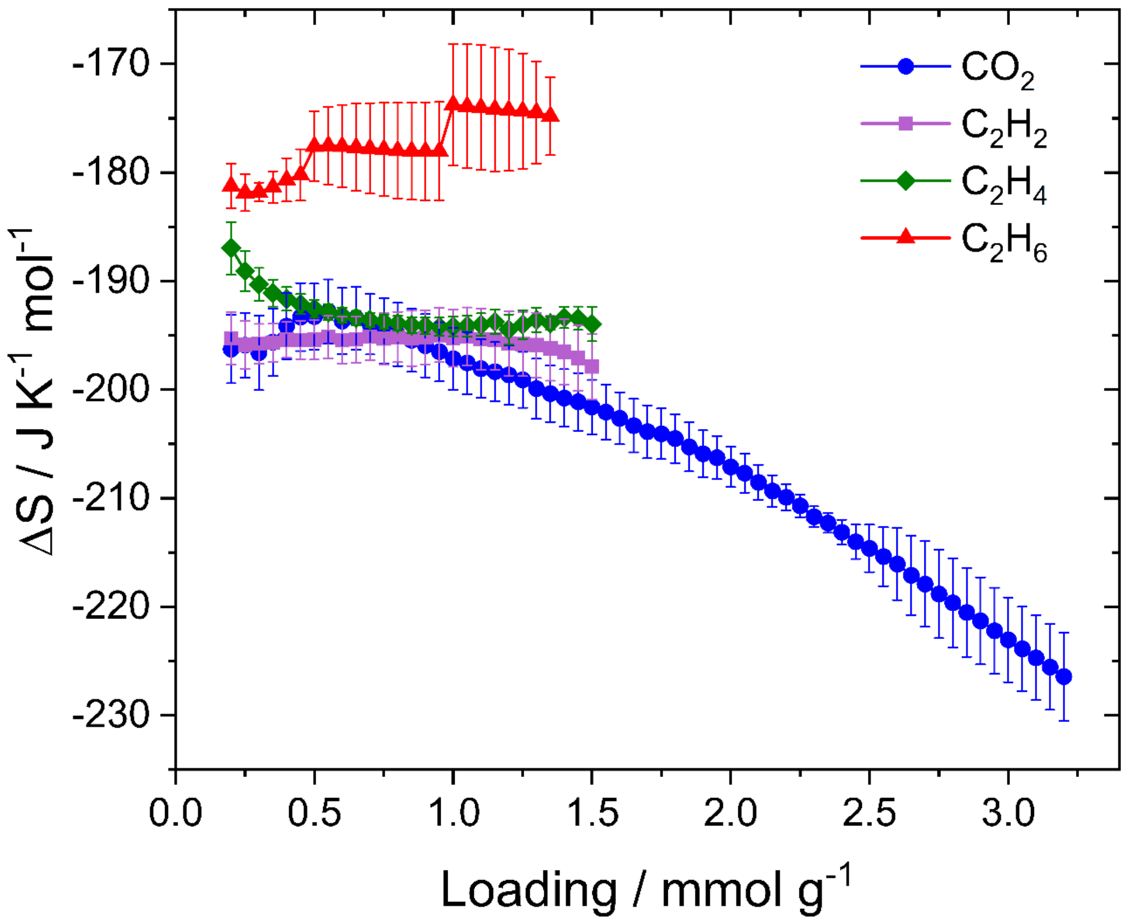

Figure S8. Entropy of adsorption (*ΔS*) for CO_2 and C_2 hydrocarbons, calculated from isothermal data for MFM-303(Al).

5. Fitting of isotherms to the dual-site Langmuir-Freundlich model

The adsorption isotherms of C_2H_2, C_2H_4, C_2H_6 and CH_4 were fitted using the dual-site Langmuir-Freundlich (DSLF) model according to the equation:

$$n = \frac{q_{sat1} b_1 P^{v_1}}{1 + b_1 P^{v_1}} + \frac{q_{sat2} b_2 P^{v_2}}{1 + b_2 P^{v_2}}$$

where n is the amount of gas adsorbed (mmol g^{-1}), q_{sati} is the saturation pressure for each site (mmol g^{-1}), b_i is the Langmuir parameter (bar^{-1}), v_i is the Freundlich parameter for each site, P is the pressure in bar. Examples of fitted isotherms are given in Figure S8.

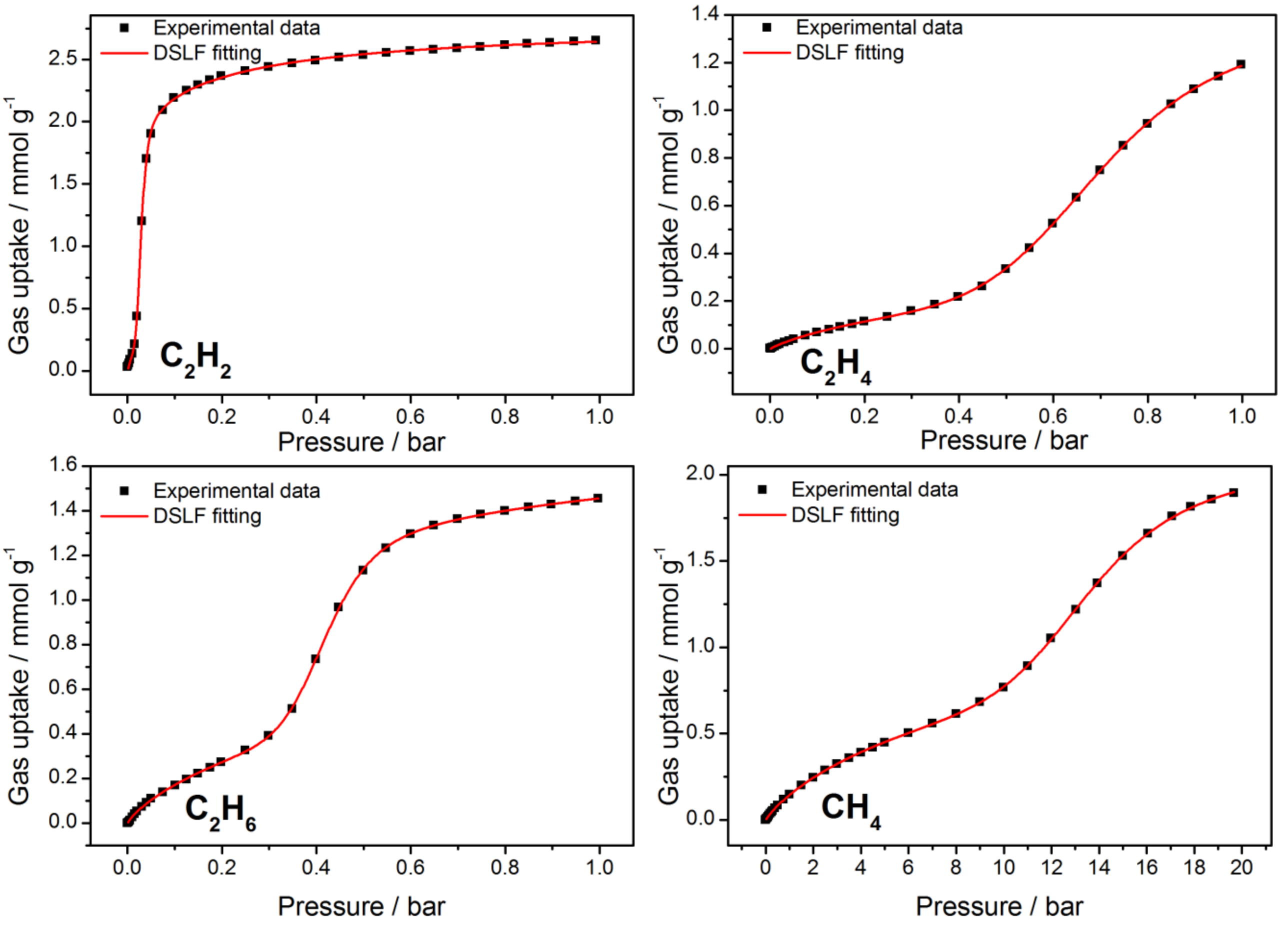

Figure S9. Example isotherms at 293 K with fits to the dual-site Langmuir-Freundlich model.

6. Analysis and derivation of the isosteric heat of adsorption for gases

The isosteric enthalpy of adsorption (*ΔH*) and entropy (*ΔS*) were calculated by fitting isotherms to the Van ‘t Hoff equation at a number of loadings:

$$\ln P = -\frac{\Delta H}{RT} + \frac{\Delta S}{R}$$

where *P* is pressure in Pa, *T* is the temperature and *R* is ideal gas constant. Linear fittings for C_2H_2, C_2H_4, CO_2, C_3H_8 gave R^2 above 0.99, with fittings for CH_4, C_2H_6 and C_3H_6 having R^2 above 0.97 indicating a good quality fit to the isotherm data.

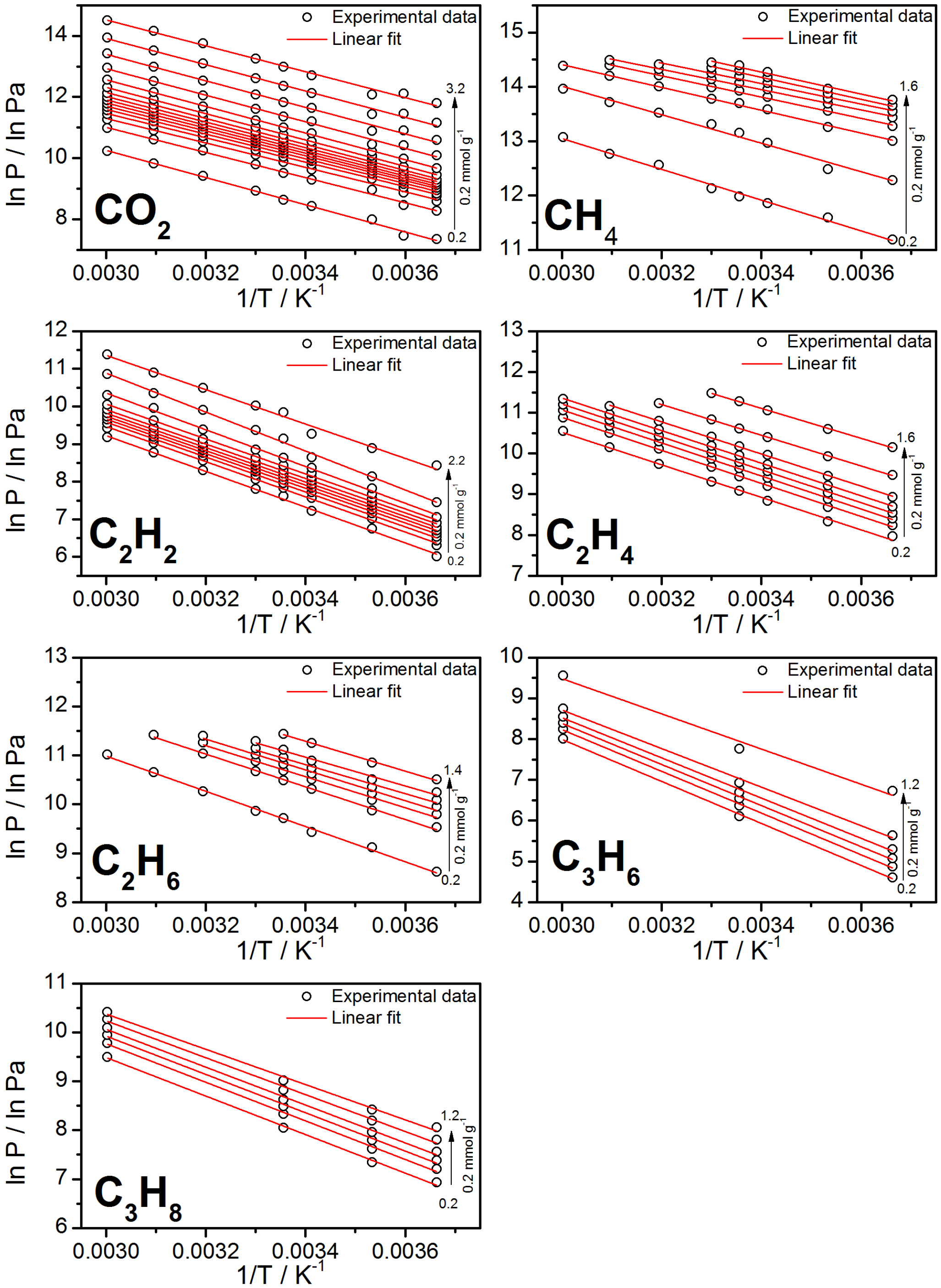

Figure S10. Linear fitting of 1/*T* vs ln *P* to determine the isosteric heat of adsorption for different gases at different loadings using the Van‘t Hoff equation.

7. IAST selectivity data

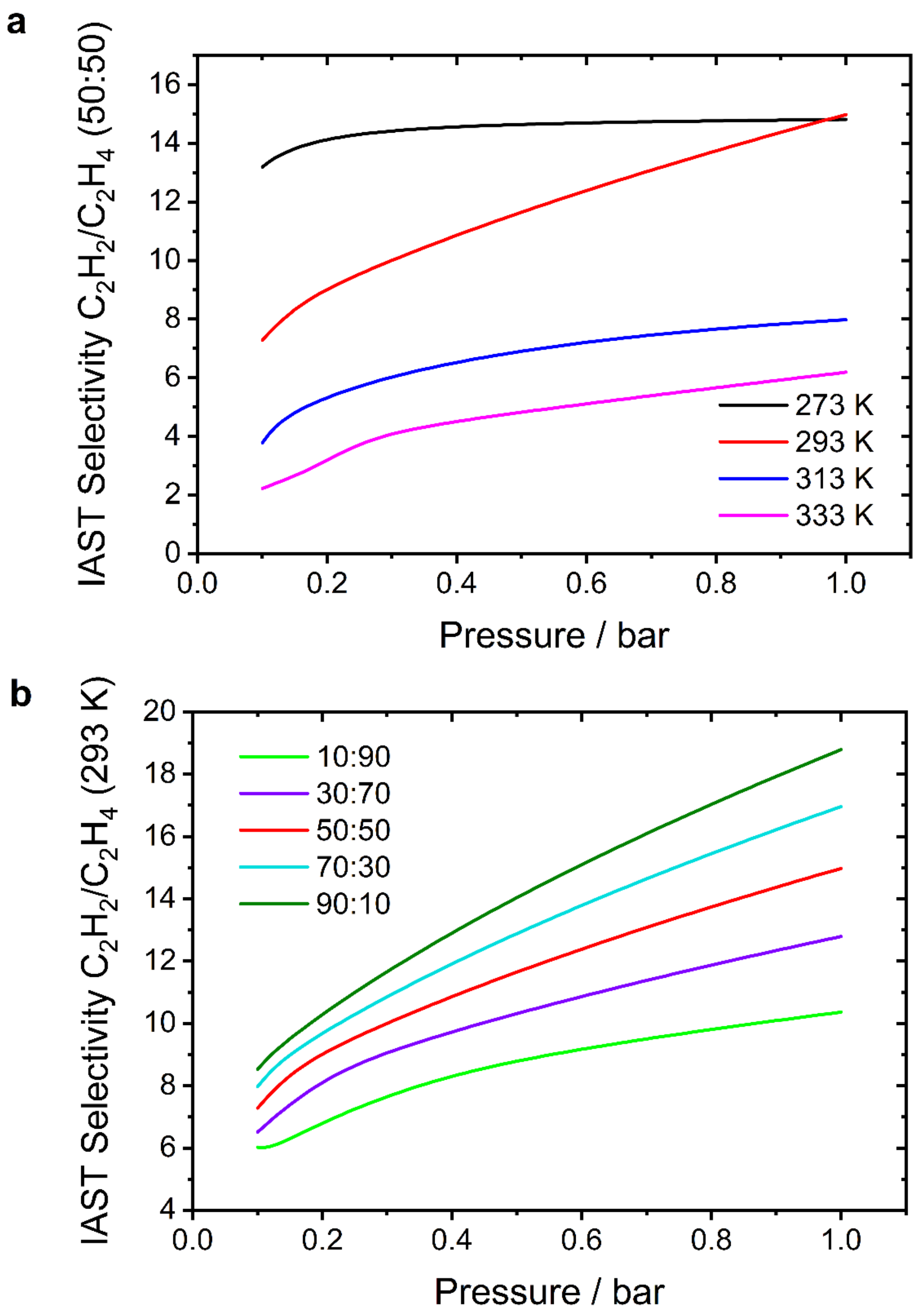

Figure S11. IAST selectivity data for C_2H_2/C_2H_4 in MFM-303(Al) at different temperatures for (a) equimolar mixtures and (b) different molar ratios at 293 K.

8. Density Functional Theory (DFT) Calculations

Modelling by Density Functional Theory (DFT) of the bare, CO_2- and C_2H_2-loaded MOFs was performed using the Vienna Ab initio Simulation Package (VASP).[2] The calculation used the Projector Augmented Wave (PAW) method[3,4] to describe the effects of core electrons, and Perdew-Burke-Ernzerhof (PBE)[5] implementation of the Generalized Gradient Approximation (GGA) for the exchange-correlation functional. Energy cutoff was 800 eV for the plane-wave basis of the valence electrons. The lattice parameters and atomic coordinates determined by synchrotron X-ray single crystal diffraction in this work were used as the initial structure. Due to the large unit cell (~300 atoms), all electronic structure and phonon calculations were performed on the Γ point only. The total energy tolerance for electronic energy minimization was 10^{-8} eV, and for structure optimization it was 10^{-7} eV. The maximum interatomic force after relaxation was below 0.001 eV/Å. The optB86b-vdW functional[6] for dispersion corrections was applied. The vibrational eigenfrequencies and modes were then calculated by solving the force constants and dynamical matrix using Phonopy.[7] The OClimax software[8,9] was used to convert the DFT-calculated phonon results to the simulated INS spectra.

9. Additional views of crystal structures

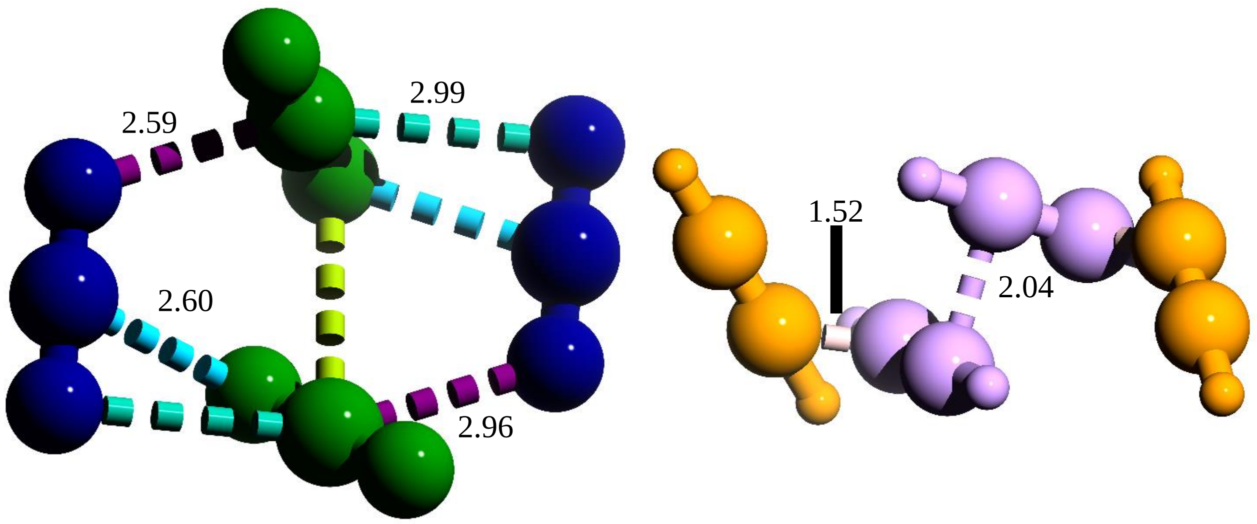

Figure S12. Views of guest-loaded MFM-303(Al) showing the intermolecular interactions between the adsorbed gas molecules at different sites. (a) CO_2 (blue, site I; green, site II), (b) C_2H_2 (orange, site I; pink, site II).

10. Comparisons to other MOFs

Table S2. Comparison of adsorption uptakes and IAST selectivities for various MOFs

	MFM-300(Al)[10.11]	Fe-MOF-74[12–14]	SIFSIX-2-Cu-i[15,16]	PCM-48[17]	UiO-66-(COOH)$_2$[18–20]	Ni-gallate[21-23]	ZJU-72[24]	NTU-72[25]	UTSA-100[26]	BSF-10[27]	NbU-7-Cl[28]	MFM-303(Al)
Surface area / m^2 g^{-1}	1370	1350	503	575	622	424	1184	315	970	426	507.8	724
Pore volume / cm^3 g^{-1}	0.375	0.626	0.26	0.17	0.21	0.18	0.635	0.415	0.399	0.216	0.709	0.194
Pore size / Å	6.5 x 6.5	11 x 11	5.2 x 5.2	~7 × ~13	4.8 x 4.8	3.5 x 4.8	4.8 x 4.8	4.3 x 4.8	4.3 x 4.3	4 x 9	4.2 x 4.2	~8 x ~2.4
CO_2 uptake at 1 bar, 273 K / mmol g^{-1}	7.0	6.47*	5.40*	1.21	3.0	7.0	6.19	0.9*	-	1.75$^{\lozenge}$	1.96	3.1
CO_2 uptake at 0.1 bar, 273 K / mmol g^{-1}	1.80	3.61*	1.72*	0.19	0.7	5.8	-	0.2*	-	0.28$^{\lozenge}$	0.44	1.84
C_2H_2 uptake at 1 bar, 293 K / mmol g^{-1}	6.34	6.8†	4.02*	1.14$^{\Delta}$	2.2*	3.64*	7.49*	1.84*	4.27$^{\Delta}$	2.9*	2.51	2.65
C_2H_4 uptake at 1 bar, 293 K / mmol g^{-1}	4.28	6.1†	2.19*	0.88$^{\Delta}$	1.8*	1.97*	-	0.97*	1.66$^{\Delta}$	1.76*	1.36	1.75
C_2H_6 uptake at 1 bar, 293 K / mmol g^{-1}	0.85	5.0†	-	0.96$^{\Delta}$	1.8*	0.28*	-	-	-	-	-	1.45
CH_4 uptake at 1 bar, 293 K / mmol g^{-1}	0.29	0.8†	0.47*	0.29$^{\Delta}$	0.29 (303 K, 1.	0.29 298 K	1.12 298 K	-	-	-	-	0.15
Selectivity C_2H_2/C_2H_4 ‡	2.30	1.87	41.0	1.9	-	43.7^	-	56^	~20	2.9	4.0	15.0
Selectivity C_2H_4/C_2H_6‡	48.7	13.6	-	-	0.9	16.8	-	-	-	-	-	2.32
Selectivity C_2H_6/CH_4‡	~5.1	~35	-	-	-	-	-	-	-	-	-	17.0
Selectivity CO_2/CH_4‡	51.6	20.2	33	6.1	-	-	6.8	-	-	-	-	56.1
Q_{st} (CO_2) # / kJ mol^{-1}	30	48-55	31.9	15.4	33.6	37	15.1	-	-	27.4	27.9	37.0
Q_{st} (C_2H_2) # / kJ mol^{-1}	32	47	41.9	23.6	38.6	46	9.7	43.5	22	34.8	36.6	39.5
Q_{st} (C_2H_4) # / kJ mol^{-1}	16	45	30.7	23.2	27.4	32	-	36.7	-	22.9	25.9	33.2
Q_{st} (C_2H_6) # / kJ mol^{-1}	11	25	-	23.4	26.8	-	-	-	-	-		30.0
Q_{st} (CH_4) # / kJ mol^{-1}	-	20	-	11.4	-	-	9.3	-	-	-	-	19

*298 K data and hence slightly higher uptakes are expected for isotherms at lower temperatures. † measured at 318 K. $^{\Delta}$ measured at 296 K. $^{\lozenge}$ measured at 278 K. ‡IAST selectivity for an equimolar mixture at 1 bar. ^IAST selectivity for a 1:99 mixture at 1 bar. #Q_{st} values at low surface coverage.

11. Physical properties of common adsorbates

Table S3. Physical properties of common adsorbates[29]

**Adsorbate**	**Kinetic diameter / Å**	**Polarizability / x 10^{-25} cm^3**	**Critical temperature / K**
CO_2	3.3	29.11	304.12
CH_4	3.758	25.93	98.60
C_2H_2	3.3	33.3-39.3	308.30
C_2H_4	4.163	42.52	282.34
C_2H_6	4.443	44.3-44.7	305.32
C_3H_6	4.687	56.6	364.90
C_3H_8	4.3-5.118	62.9-63.7	369.83

12. Supplementary references

1 S. Yang, J. Sun, A. J. Ramirez-Cuesta, S. K. Callear, W. I. F. David, D. P. Anderson, R. Newby, A. J. Blake, J. E. Parker, C. C. Tang and M. Schröder, *Nat. Chem.*, 2012, **4**, 887–894.

2 G. Kresse and J. Furthmüller, *Phys. Rev. B*, 1996, **54**, 11169–11186.

3 P. E. Blöchl, *Phys. Rev. B*, 1994, **50**, 17953–17979.

4 G. Kresse and D. Joubert, *Phys. Rev. B*, 1999, **59**, 1758–1775.

5 J. P. Perdew, K. Burke and M. Ernzerhof, *Phys. Rev. Lett.*, 1996, **77**, 3865–3868.

6 J. Klimeš, D. R. Bowler and A. Michaelides, *J. Phys. Condens. Matter*, 2010, **22**, 022201.

7 A. Togo and I. Tanaka, *Scr. Mater.*, 2015, **108**, 1–5.

8 Y. Q. Cheng, L. L. Daemen, A. I. Kolesnikov and A. J. Ramirez-Cuesta, *J. Chem. Theory Comput.*, 2019, **15**, 1974–1982.

9 Y. Cheng, L. L. Daemen, A. I. Kolesnikov and A. J. Ramirez-Cuesta, OCLIMAX, Oak Ridge National Laboratory, Oak Ridge, Tennessee 2019.

10 S. Yang, J. Sun, A. J. Ramirez-Cuesta, S. K. Callear, W. I. F. David, D. P. Anderson, R. Newby, A. J. Blake, J. E. Parker, C. C. Tang and M. Schröder, *Nat. Chem.*, 2012, **4**, 887–894.

11 S. Yang, A. J. Ramirez-Cuesta, R. Newby, V. Garcia-Sakai, P. Manuel, S. K. Callear, S. I. Campbell, C. C. Tang and M. Schröder, *Nat. Chem.*, 2014, **7**, 121–129.

12 S. J. Geier, J. A. Mason, E. D. Bloch, W. L. Queen, M. R. Hudson, C. M. Brown and J. R. Long, *Chem. Sci.*, 2013, **4**, 2054–2061.

13 E. D. Bloch, W. L. Queen, R. Krishna, J. M. Zadrozny, C. M. Brown and J. R. Long, *Science*, 2012, **335**, 1606–1610.

14 W. Lou, J. Yang, L. Li and J. Li, *J. Solid State Chem.*, 2014, **213**, 224–228.

15 X. Cui, K. Chen, H. Xing, Q. Yang, R. Krishna, Z. Bao, H. Wu, W. Zhou, X. Dong, Y. Han, B. Li, Q. Ren, M. J. Zaworotko and B. Chen, *Science*, 2016, **353**, 141–144.

16 P. Nugent, Y. Belmabkhout, S. D. Burd, A. J. Cairns, R. Luebke, K. Forrest, T. Pham, S. Ma, B. Space, L. Wojtas, M. Eddaoudi and M. J. Zaworotko, *Nature*, 2013, **495**, 80–84.

17 J. E. Reynolds, K. M. Walsh, B. Li, P. Kunal, B. Chen and S. M. Humphrey, *Chem. Commun.*, 2018, **54**, 9937–9940.

18 J. M. Taylor, K. W. Dawson and G. K. H. Shimizu, *J. Am. Chem. Soc.*, 2013, **135**, 1193–1196.

19 L. Zhang, L. Li, E. Hu, L. Yang, K. Shao, L. Yao, K. Jiang, Y. Cui, Y. Yang, B. Li, B. Chen and G. Qian, *Adv. Sci.*, 2020, **7**, 1–7.

20 L. Zhang, K. Jiang, L. Yang, L. Li, E. Hu, L. Yang, K. Shao, H. Xing, Y. Cui, Y. Yang, B. Li, B. Chen and G. Qian, *Angew. Chemie Int. Ed.*, 2021, **60**, 15995–16002.

21 J. Wang, L. Li, L. Guo, Y. Zhao, D. Xie, Z. Zhang, Q. Yang, Y. Yang, Z. Bao and Q. Ren, *Chem. – A Eur. J.*, 2019, **25**, 15516–15524.

22 F. Chen, D. Lai, L. Guo, J. Wang, P. Zhang, K. Wu, Z. Zhang, Q. Yang, Y. Yang, B. Chen, Q. Ren and Z. Bao, *J. Am. Chem. Soc.*, 2021, **143**, 9040–9047.

23 Z. Bao, J. Wang, Z. Zhang, H. Xing, Q. Yang, Y. Yang, H. Wu, R. Krishna, W. Zhou, B. Chen and Q. Ren, *Angew. Chemie Int. Ed.*, 2018, **57**, 16020–16025.

24 X. Duan, R. Song, J. Yu, H. Wang, Y. Cui, Y. Yang, B. Chen and G. Qian, *RSC Adv.*, 2014, **4**, 36419.

25 Y. Duan, Y. Huang, C. Wang, Q. Wang, K. Ge, Z. Lu, H. Wang, J. Duan, J. Bai and W. Jin, *Chem. Sci.*, 2023, **14**, 4605–4611.

26 T.-L. Hu, H. Wang, B. Li, R. Krishna, H. Wu, W. Zhou, Y. Zhao, Y. Han, X. Wang, W. Zhu, Z. Yao, S. Xiang and B. Chen, *Nat. Commun.*, 2015, **6**, 7328.

27 W. Sun, Y. Jin, Y. Wu, W. Lou, Y. Yuan, S. Duttwyler, L. Wang and Y. Zhang, *Inorg. Chem. Front.*, 2022, **9**, 5140–5147.

28 J. Wu, Y. Wang, J. P. Xue, D. Wu and J. Li, *Inorg. Chem.*, 2023, **62**, 19997–20004.

29 J. Lee, O. K. Farha, J. Roberts, K. A. Scheidt, S. T. Nguyen and J. T. Hupp, *Chem Soc Rev*, 2009, **38**, 1450–1459.
